# Potent 3CLpro inhibitors effective against SARS-CoV-2 and MERS-CoV in animal models by therapeutic treatment

**DOI:** 10.1128/mbio.02878-23

**Published:** 2023-12-21

**Authors:** Pengfei Li, Yunjeong Kim, Chamandi S. Dampalla, Harry Nhat Nguyen, David K. Meyerholz, David K. Johnson, Scott Lovell, William C. Groutas, Stanley Perlman, Kyeong-Ok Chang

**Affiliations:** 1 Department of Microbiology and Immunology, The University of Iowa, lowa, USA; 2 Department of Diagnostic Medicine and Pathobiology, College of Veterinary Medicine, Kansas State University, Manhattan, Kansas, USA; 3 Department of Chemistry, Wichita State University, Wichita, Kansas, USA; 4 Department of Pathology, The University of Iowa, Iowa, USA; 5 Computational Chemical Biology Core, The University of Kansas, Lawrence, Kansas, USA; 6 Protein Structure Laboratory, The University of Kansas, Lawrence, Kansas, USA; Virginia Polytechnic Institute and State University, Blacksburg, Virginia, USA; Virginia Polytechnic Institute and State University, Blacksburg, Virginia, USA

**Keywords:** protease inhibitors, 3CLpro, SARS-CoV-2, MERS-CoV, animal models

## Abstract

Severe acute respiratory syndrome coronavirus 2 (SARS-CoV-2) and Middle East respiratory syndrome coronavirus (MERS-CoV) are zoonotic betacoronaviruses that continue to have a significant impact on public health. Timely development and introduction of vaccines and antivirals against SARS-CoV-2 into the clinic have substantially mitigated the burden of COVID-19. However, a limited or lacking therapeutic arsenal for SARS-CoV-2 and MERS-CoV infections, respectively, calls for an expanded and diversified portfolio of antivirals against these coronavirus infections. In this report, we examined the efficacy of two potent 3CLpro inhibitors, ***5d*** and ***11d***, in fatal animal models of SARS-CoV-2 and MERS-CoV to demonstrate their broad-spectrum activity against both viral infections. These compounds significantly increased the survival of mice in both models when treatment started 1 day post infection compared to no treatment which led to 100% fatality. Especially, the treatment with compound ***11d*** resulted in 80% and 90% survival in SARS-CoV-2 and MERS-CoV-infected mice, respectively. Amelioration of lung viral load and histopathological changes in treated mice correlated well with improved survival in both infection models. Furthermore, compound ***11d*** exhibited significant antiviral activities in K18-hACE2 mice infected with SARS-CoV-2 Omicron subvariant XBB.1.16. The results suggest that these are promising candidates for further development as broad-spectrum direct-acting antivirals against highly virulent human coronaviruses.

**IMPORTANCE:**

Human coronaviruses such as severe acute respiratory syndrome coronavirus 2 (SARS-CoV-2) and Middle East respiratory syndrome coronavirus (MERS-CoV) continue to have a significant impact on public health. A limited or lacking therapeutic arsenal for SARS-CoV-2 and MERS-CoV infections calls for an expanded and diversified portfolio of antivirals against these coronavirus infections. We have previously reported a series of small-molecule 3C-like protease (3CLpro) inhibitors against human coronaviruses. In this report, we demonstrated the *in vivo* efficacy of 3CLpro inhibitors for their broad-spectrum activity against both SARS-CoV-2 and MERS-CoV infections using the fatal animal models. The results suggest that these are promising candidates for further development as broad-spectrum direct-acting antivirals against highly virulent human coronaviruses.

## INTRODUCTION

Members of the family *Coronaviridae* are enveloped, single-stranded, positive-sense RNA viruses and include important human pathogens, such as severe acute respiratory syndrome coronavirus (SARS-CoV), severe acute respiratory syndrome coronavirus-2 (SARS-CoV-2), and Middle East respiratory syndrome coronavirus (MERS-CoV) (1). The most recently emerged coronavirus, SARS-CoV-2, the causative agent of COVID-19, continues to have a huge impact on public health globally (2–4). Timely development and introduction of effective vaccines and antivirals into the clinic have mitigated the impact and threat of COVID-19 (5, 6). However, rapidly arising SARS-CoV-2 variants calls for an expanded and diversified portfolio of antivirals, such as direct-acting antivirals (DAAs) against SARS-CoV-2 variants, as well as emerging and re-emerging coronaviruses, including MERS-CoV (4, 7–12).

The coronavirus RNA genome encodes non-structural and structural proteins (1). Non-structural proteins are expressed as two large polyproteins (designated pp1a and pp1ab) which are processed by two viral proteases: 3C-like protease (3CLpro) and papain-like protease (PLpro) (1). Coronavirus 3CLpro is responsible for the majority of cleavages within viral polyproteins, and coronavirus 3CLpros are highly conserved with a unique primary substrate specificity for a P1 Gln residue (13, 14). The 3CLpro has proven to be a validated therapeutic target for coronavirus diseases of humans and animals with examples of Nirmatrelvir for COVID-19 (15) and GC376 for feline infectious peritonitis, a fatal coronavirus disease in cats (16). Several 3CLpro inhibitors have been shown to be effective in experimentally infected (mice and hamsters) as a single or combination treatment for SARS-CoV-2 infection (15, 17–27) or MERS-CoV infection (28).

Multiple host proteases have been implicated in the activation of SARS-CoV-2 spike protein (S), which is essential in the entry of virus (29–31). Because of the roles of proteases, including furin, trypsin, and transmembrane protease serine 2 (TMPRSS2), in the activation of SARS-CoV-2 S protein, inhibitors of these host proteases have been explored as entry-blocking antivirals (29, 31, 32). The alternative endosomal entry route of SARS-CoV-2 is dependent on the activation of SARS-CoV-2 S protein by cathepsin enzymes (33, 34). Some inhibitors of SARS-CoV-2 3CLpro, including those from our lab (28, 35–38), have dual inhibitory effects against cathepsins (28, 33, 39, 40). In this report, we further examined selected compounds (***5c/d*** and ***11c/d***) from our previous report (41) for the potential dual roles of the inhibitors against 3CLpro and cathepsins in virus replication using lentivirus-based pseudotyped viruses expressing coronavirus S (42). We then examined the *in vivo* efficacy of compounds ***5d*** and ***11d*** using animal models of SARS-CoV-2 and MERS-CoV using BALB/c and human dipeptidyl peptidase 4 (hDDP4) knock-in (KI) mice, respectively. Moreover, the efficacy of compound ***11d*** was tested in K18-hACE2 mice infected with SARS-CoV-2 Omicron subvariant XBB.1.16.

## RESULTS

### Activity of *5c/d* and *11c/d* on SARS-CoV-2 pseudovirus entry assays in cells

The efficacy of ***5c/d*** and ***11c/d*** against 3CLpros of SARS-CoV-2 and MERS-CoV in enzyme assays and SARS-CoV-2 replication and pseudovirus entry assay in cells expressing hACE2 or hACE2 plus TMPRSS2 is summarized in Table 1. The potency of ***5c/d** and **11c/d*** against 3CLpros of SARS-CoV-2 and MERS-CoV or SARS-CoV-2 replicon was high with nanomolar IC_50_ and EC_50_ values and was reported in our previous publication (41). The EC_50_ values of ***5c/d** and **11c/d*** against the entry of SARS-CoV-2 pseudoviruses, and all data from MDL28170 and Nafamostat were newly generated for this study. As expected, MDL28170 and Nafamostat did not have activity against the 3CLpros of SARS-CoV-2 and MERS-CoV. In terms of virus entry, ***5c/d** and **11c/d**,* and MDL28170 strongly inhibited the entry of pseudotyped viruses into cells expressing hACE2 but not in cells expressing hACE2 plus TMPRSS2. In contrast, Nafamostat was highly potent at inhibiting pseudovirus entry in cells expressing both hACE2 and TMPRSS2 with an EC_50_ 0.001 µM, but it had little effect in cells expressing hACE2 alone at up to 50 µM, as expected for a TMPRSS2 inhibitor (Table 1).

**TABLE 1:** Activities of ***5c/d** and **11c/d*** in enzyme assays (SARS-CoV-2 3CLpro, MERS-CoV 3CLpro, and cathepsin L) and cell-based assays using SARS-CoV-2 replicon or SARS-CoV-2 pseudoviruses in cells expressing hACE2 or hACE2 plus TMPRSS2

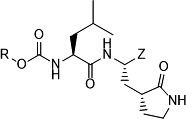							
Compounds	R	Z	IC_50_ (µM)^*a*^		EC_50_ (µM)		
			SARS-CoV-2	MERS-CoV	SARS-CoV-2 replicon^*a*^	Pseudovirus entry assay	
						hACE2	hACE2+ TMPRSS2
5c	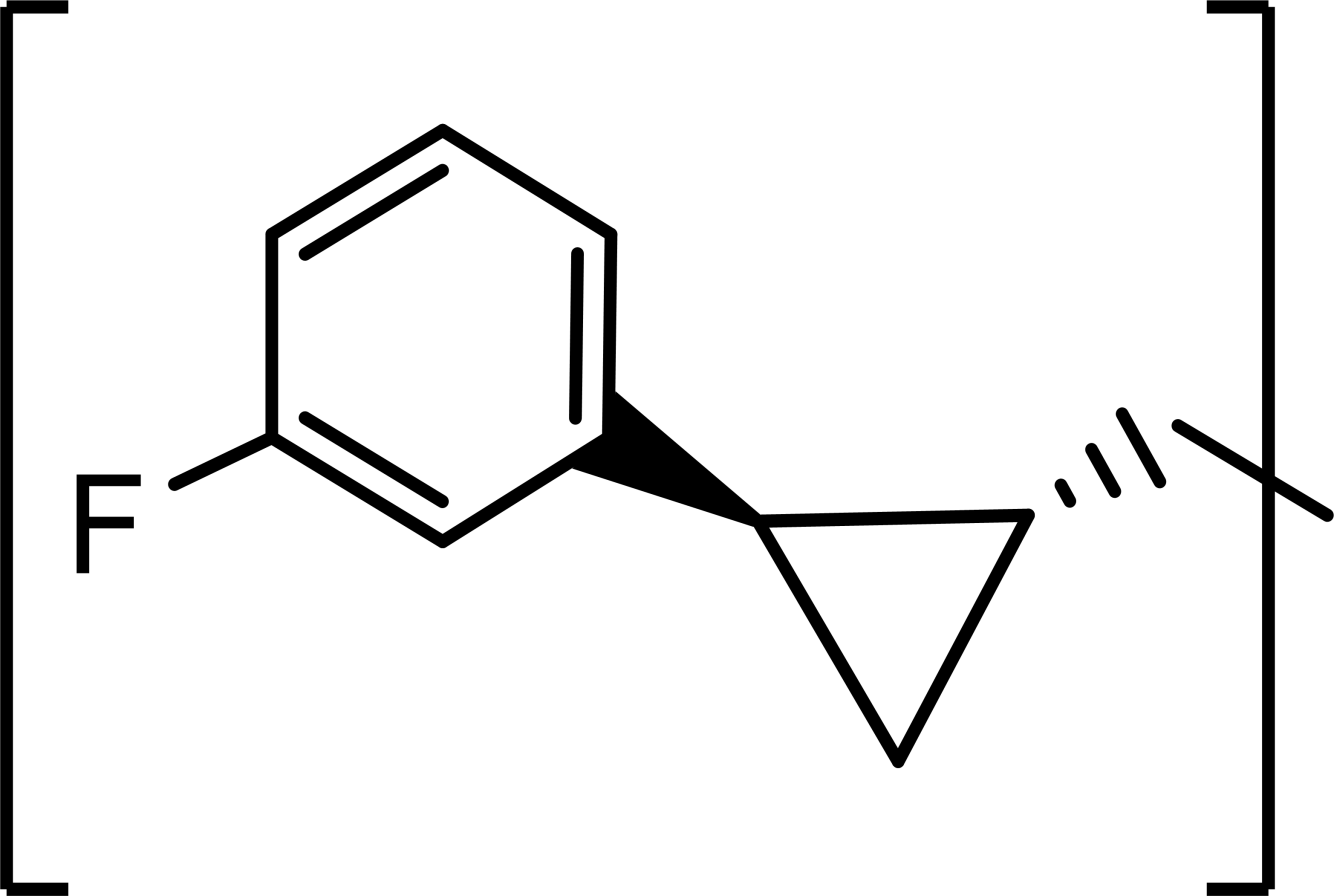	-CHO	0.18 ± 0.03^*b*^	0.08 ± 0.03	0.012 ± 0.06	0.06 ± 0.03	>50
5d		-CH(OH)SO_3_Na	0.19 ± 0.02	0.07 ± 0.03	0.013 ± 0.05	0.05 ± 0.02	>50
11c	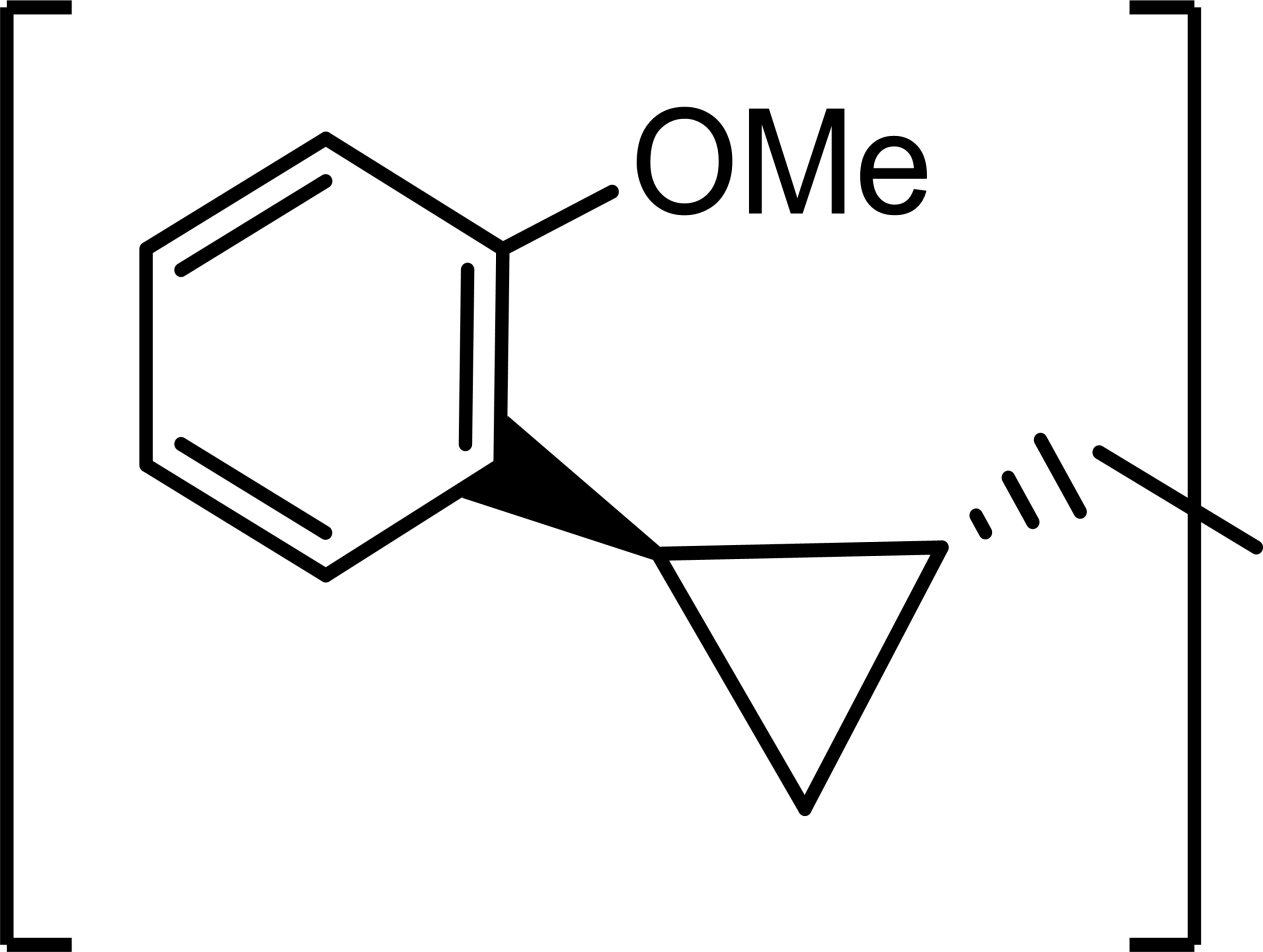	-CHO	0.14 ± 0.04	0.12 ± 0.11	0.011 ± 0.06	0.13 ± 0.03	>50
11d		-CH(OH)SO_3_Na	0.14 ± 0.05	0.07 ± 0.01	0.012 ± 0.07	0.12 ± 0.05	>50
MDL28170	N/A^*c*^	N/A	>50	>50	>50	0.01 ± 0.03	>50
Nafamostat	N/A	N/A	>50	>50	>50	>50	0.005 ± 0.02

^*a*^ Values for 5c/d and 11c/d were previously published (41).

^*b*^ values are averages ± standard deviations.

^*c*^ N/A, Not applicable.

### Activity of 5c/d and 11c/d against human proteases

Inhibitors ***5c/d** and **11c/d*** potently inhibited cathepsins L and B with IC_50_ values of 0.02–0.03 and 0.23–0.31 µM, respectively (Table 2). However, these compounds had little activity against thrombin, chymotrypsin, neutrophile elastase, cathepsin D or G at up to 50 µM (Table 2).

**TABLE 2:** Activities of ***5c/d** and **11c/d*** against human proteases including thrombin, chymotrypsin, and cathepsins B, D, G, and L in enzyme assays

Compounds	IC_50_ (µM)^*a*^						
	Thrombin	Chymotrypsin	Neutrophilelastase	Cat B	Cat D	Cat G	Cat L
5c	>50	>50	>50	0.24 ± 0.05	>50	>50	0.03 ± 0.01
5d	>50	>50	>50	0.23 ± 0.08	>50	>50	0.02 ± 0.02
11c	>50	>50	>50	0.31 ± 0.05	>50	>50	0.03 ± 0.01
11d	>50	>50	>50	0.27 ± 0.06	>50	>50	0.02 ± 0.02

^*a*^ Values are averages ± standard deviations.

### Modelling of the *11c* binding mode with SARS-CoV-2 and MERS-CoV 3CLpro

**Fig 1:**
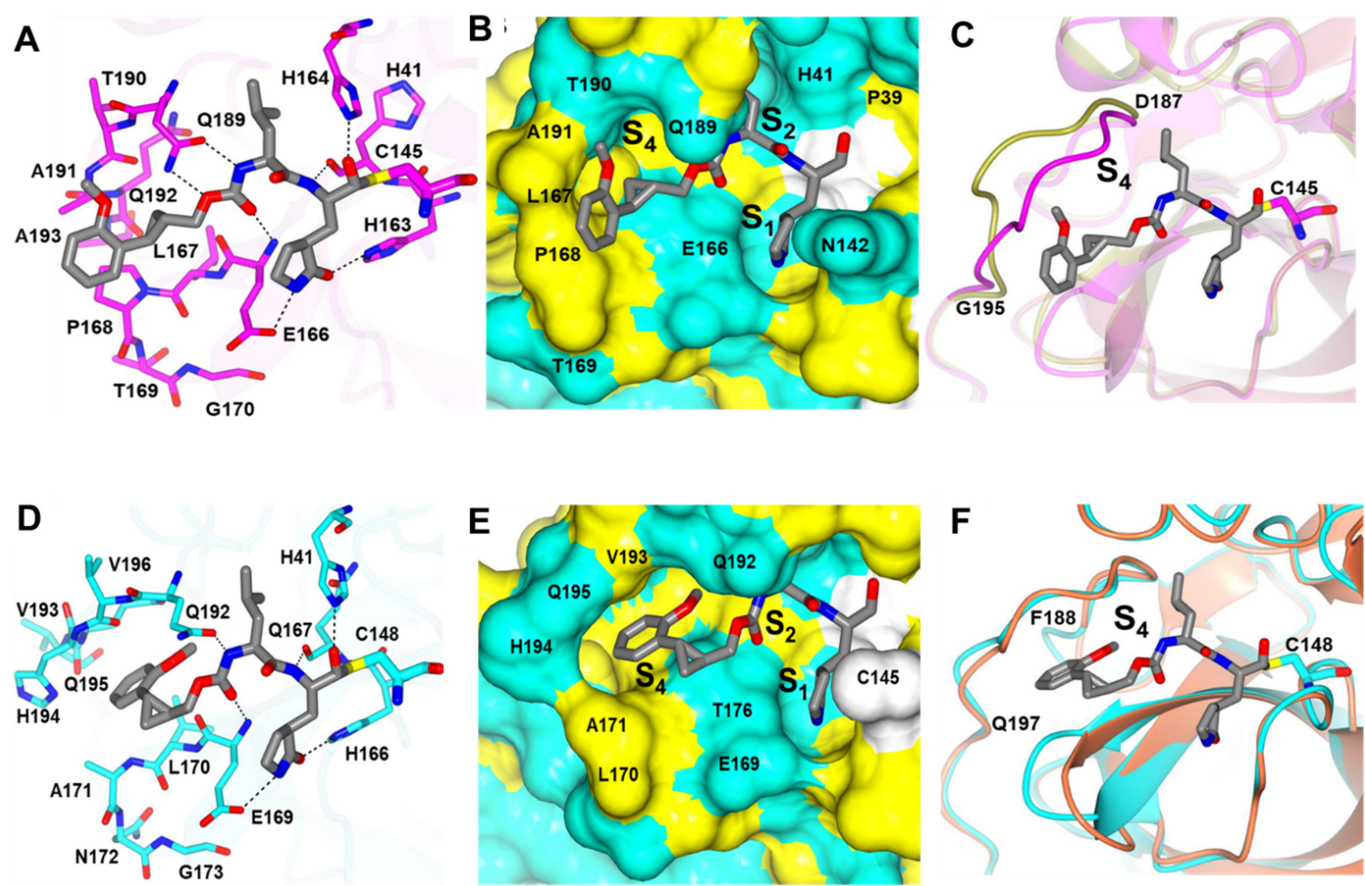
Modelling of *11*c in the active sites of SARS-CoV-2 3CLpro (**A–C**) and MERS-CoV 3CLpro (**D–F**). (A, D) Hydrogen bond interactions (dashed lines). (B, E) Surface representation showing the putative orientation of the *o*-methoxyphenyl ring in the S4 subsite. Neighboring residues are colored yellow (non-polar), cyan (polar), and white (weakly polar). (C, F). Superposition of inhibitor-bound SARS-CoV-2 3CLpro (magenta) or MERS-CoV 3CLpro (blue) with that apo protein structure (PDB 7JST, gold) showing the differences in the loop (D187-G195 for C) or (F188-197 for F) near the S4 subsite.

Compound *11*c adopts a conformation in the SARS-CoV-2 3CLpro active site where the side chains of the glutamine surrogate and Leu residues of the inhibitor are positioned with the S1 and S2 pockets, respectively, resulting in similar hydrogen bond interactions typically observed in the crystal structures (Fig. 1A). The orientation of the *o*-methoxylphenyl ring is positioned near the hydrophobic residues in the S4 subsite with the methoxy group directed towards the surface (Fig. 1B). Superposition (43) of the *11*c bound structure with the apo structure of SARS-CoV-2 3CLpro is similar overall with an RMSD deviation between Cα atoms of 0.75 Å (298 residues). The main differences are observed in the loop spanning D187-G195 near the S4 subsite which moves toward the inhibitor to form a hydrogen bond with Q189 and also engage in hydrophobic interactions with the inhibitor (Fig. 1C). The binding mode of *11*c in the active site of MERS 3CLpro adopts a similar binding mode relative to SARS-CoV-2 3CLpro, as shown in Fig. 1D. The main difference is that the *o*-methoxyphenyl ring is positioned deep into the S4 pocket although no hydrogen bonds are formed with the methoxy group (Fig. 1E). Although there are no apo MERS 3CLpro structures available to our knowledge, the crystal structure of the catalytically inactive mutant (C148A) has been determined in the apo form. Superposition indicated a high degree of structural similarity with RMSD deviation between Cα atoms of 0.75 Å (297 residues). Unlike SARS-CoV-2 3CLpro, the loop near the S4 subsite (F188-Q197) is similar in both structures (Fig. 1F).

### Survival and morbidity in infected mice

**Fig 2:**
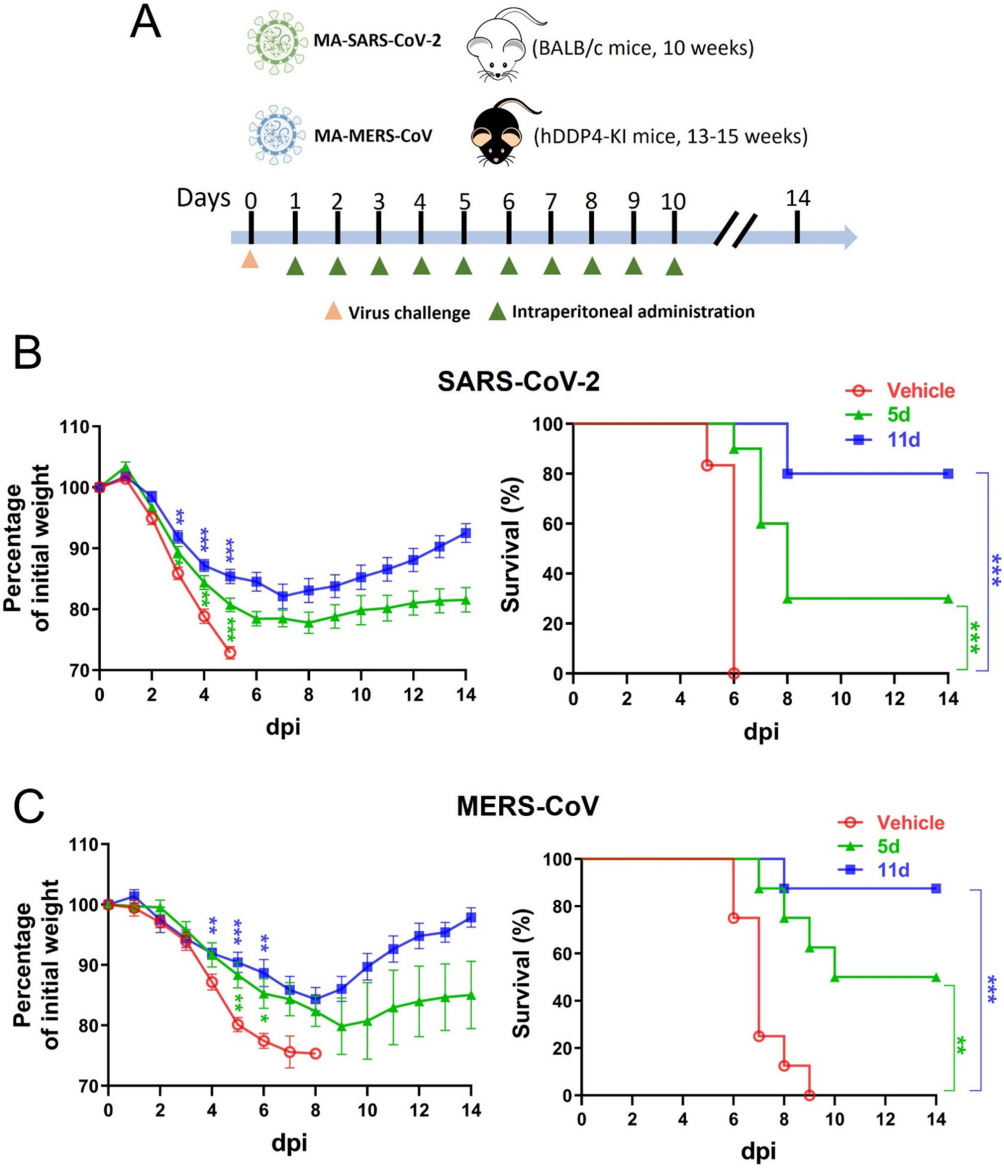
Post-infection treatment of ***5d*** or ***11d*** in BALB/c mice infected with MA-SARS-CoV-2 or the hDPP4-KI mice infected with MA-MERS-CoV. (**A**) Schematic of the experiment design for testing the therapeutic effect of *5d* and *11d* on viruses in mice. (**B**) The BALB/c mice were infected with MA-SARS-CoV-2 (*N* = 10) at 0 dpi and treated with ***5d*** or ***11d*** for 10 days starting at 1 dpi, and body weight (B, left) and survival (B, right) were monitored for 14 days. (**C**) The hDPP4-KI mice were infected with MA-MERS-CoV (*N* = 10) at 0 dpi and treated with ***5d*** or ***11d*** for 10 days starting at 1 dpi, and body weight (C, left) and survival (C, right) were monitored for 14 days. Control mice received the vehicle in both animal studies. Data are presented as mean ± standard error of mean (SEM). Asterisks represent statistical significance between vehicle-treated group and ***5d***- or ***11d***-treated group (*P  < 0.05, **P  < 0.01, ***P < 0.001) (Blue and green asterisks indicate comparison between vehicle- and ***11d** or **5d**-*treated groups, respectively.

Compounds ***5d*** and ***11d*** were tested in mouse models of SARS-CoV-2 or MERS-CoV infection because both compounds had high potency in the enzyme and cell-based assays. In mice infected with mouse-adapted (MA) SARS-CoV-2 (SARS2-N501Y_MA30_) or MA-MERS-CoV, treatment with vehicle led to 100% fatality by 6 or 9 days after virus infection, respectively (Fig. 2B and C). However, compound ***11d*** treatment led to the survival of 80% or 90% of mice infected with MA-SARS-CoV-2 or MA-MERS-CoV, respectively (Fig. 2B and C). Treatment with compound ***5d*** resulted in 30% or 50% survival of MA-SARS-CoV-2 or MA-MERS-CoV -infected mice, respectively. Mice treated with vehicle lost significant body weight following MA-SARS-CoV-2 or MA-MERS-CoV infection before they were euthanized (Fig. 2B and C). Less weight loss was observed in mice treated with compound ***5d*** or ***11d*** compared to vehicle-treated mice (Fig. 2B and C). After reaching a nadir at 7–9 dpi, surviving mice treated with ***5d*** or ***11d*** gradually gained body weight, but weight gain was greater in those treated with ***11d*** compared to ***5d*** treatment (Fig. 2B and C).

### Viral loads and lung pathology in animal studies

**Fig 3:**
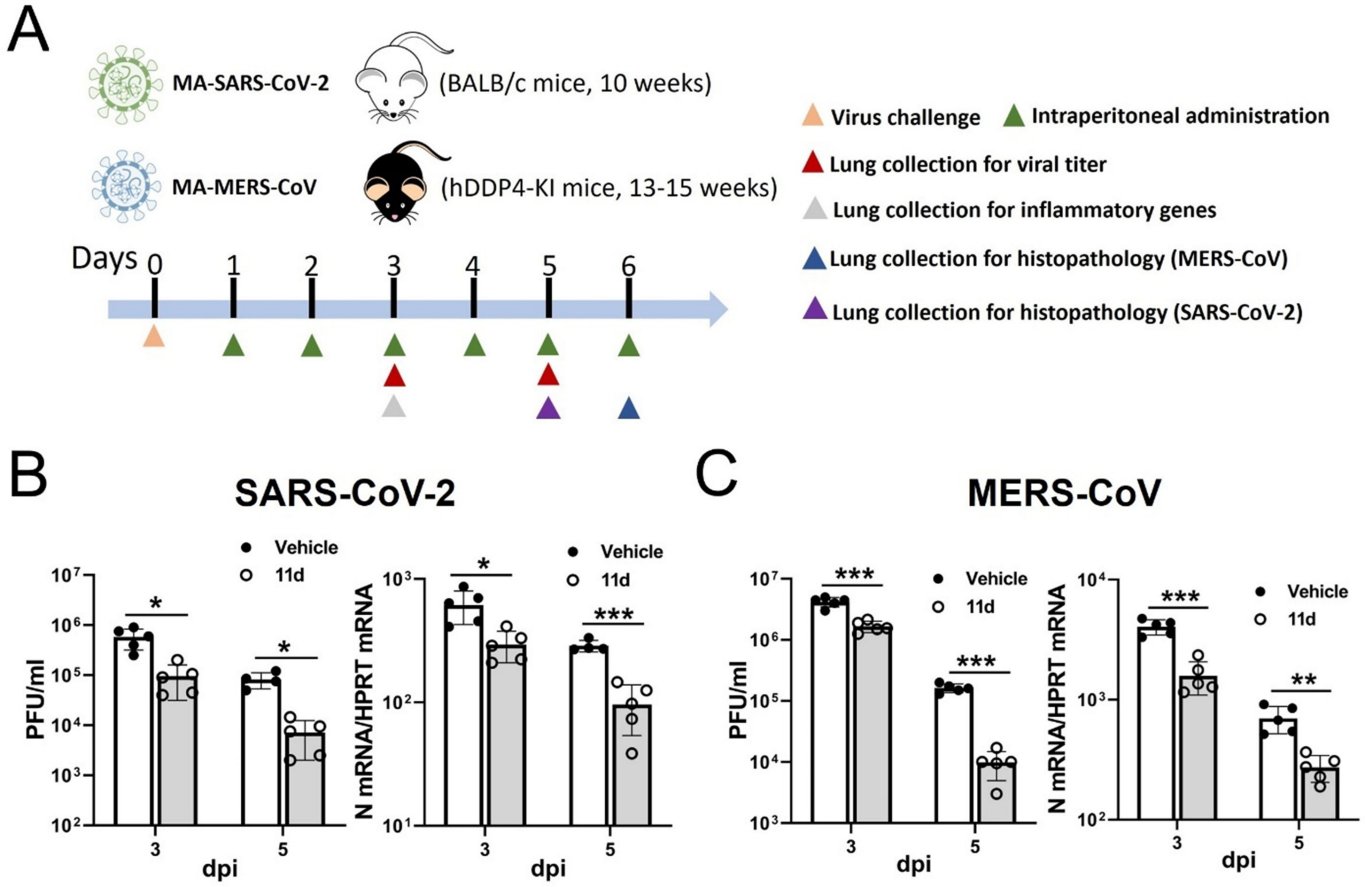
Lung virus titers of BALB/c or hDPP4-KI mice infected with MA-SARS-CoV-2 or MA-MERS-CoV, respectively. (**A**) Schematic of experimental design for Fig. 3 and 4. (**B**) The mice were infected with the MA-SARS-CoV-2 or MA-MERS-CoV at 0 dpi and treated with vehicle or compound ***11d*** starting at 1 dpi. Lungs were collected for virus titration (B, left and C, left) or viral N gene quantitation (B, right and C, right) at 3 and 5 dpi of SARS-CoV-2 or MERS-CoV infection, respectively. Data are presented as mean ± SEM. Asterisks indicate statistical significance (*P < 0.05, **P < 0.01, ***P < 0.001).

**Fig 4:**
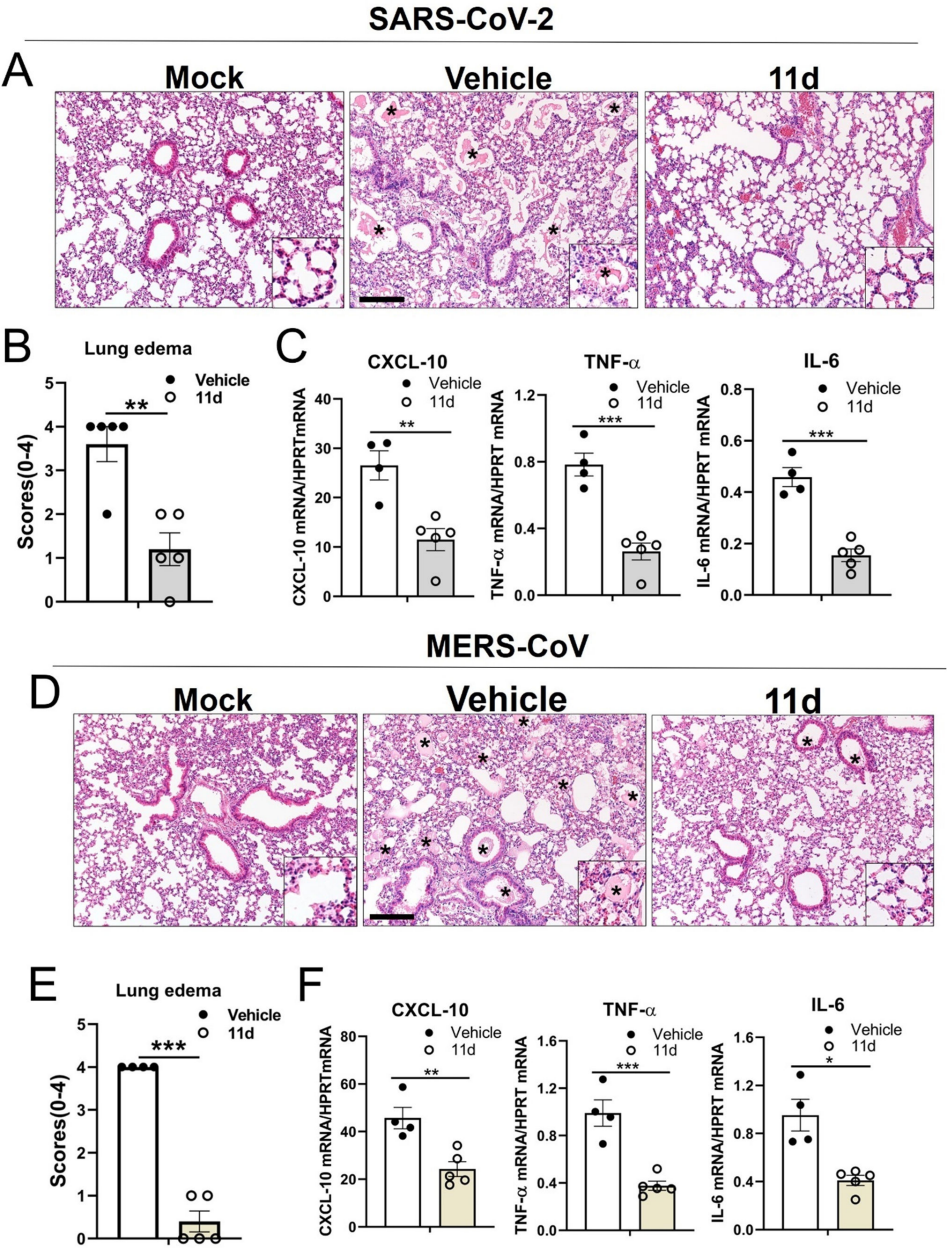
Lung histopathology of BALB/c or hDPP4-KI mice infected with MA-SARS-CoV-2 or MA-MERS-CoV, respectively. As shown in Fig. 3A, the mice were infected with MA-SARS-CoV-2 or MA-MERS-CoV at 0 dpi and treated with vehicle or ***11d*** starting at 1 dpi. Lungs were collected for histopathologic examination at 5 dpi (MA-SARS-CoV-2) (**A**) or 6 dpi (MA-MERS-CoV) (**D**). Average scores of lung edema by vehicle or ***11d*** treatment are shown in (**B**) and (**E**). Bar indicates 170 µm. Inflammatory genes in MA-SARS-CoV-2 (**C**) or MA-MERS-CoV (**F**) infected mice lungs were quantified at 3 dpi. Data are presented as mean ± standard error of mean (SEM). Asterisks in indicated panels represent statistical significance (*P < 0.05, **P < 0.01, ***P < 0.001).

We used compound ***11d*** for the evaluation of viral load and histopathological changes in the lungs of mice infected with MA-SARS-CoV-2 or MA-MERS-CoV. Lung virus titers peaked at 3 dpi and decreased at 5 dpi in both animal models with vehicle or ***11d*** treatment. For MA-SARS-CoV-2-infected mice, virus titers were statistically lower in ***11d**-*treated mice compared to vehicle-treated mice at 3 and 5 dpi by up to 10-fold on both days (Fig. 3B, left). Levels of SARS-CoV-2 N gene in the lungs confirmed the results of lung virus titers in both vehicle or ***11d*** treatment mice (Fig. 3B, right). Similar to SARS-CoV-2 infection, ***11d*** treatment of MA-MERS-CoV-infected mice showed that virus titers and MERS-CoV N protein levels were significantly reduced compared to vehicle-treated mice at 3 and 5 dpi (Fig. 3C). In both animal models, lung pathology included diffuse alveolar damage with progressive alveolar or interstitial lesions characterized by edema, inflammation and focal cytomegaly in some alveolar lining cells. Additional features include an accumulation of immune effector cells, including granulocytes and macrophages, evidence of cell death, hemorrhage, hyaline membranes, and occasional vascular thrombi (Fig. 4A and D). While severe edema (average score 3.6 for SARS-CoV-2 and 4 for MERS-CoV) and perivascular infiltrates were evident in the lungs from vehicle-treated animals, ***11d*** treatment led to significantly reduced lung pathologies including milder edema (average score 1 for MA-SARS-CoV-2 and 0.5 for MA-MERS-CoV) (Fig. 4A,B, D,andE). When pro-inflammatory cytokines including CXCL-10, IL-6 and TNF-α, were assessed at 3 dpi in both animal studies. The 11d treatment of MA-SARS-CoV-2 or MA-MERS-CoV infected mice showed significantly lower expression of these inflammatory genes compared to vehicle-treated mice (Fig. 4C and F).

### Post-infection treatment of *11d* protects K18-hACE2 mice against Omicron XBB.1.16 infection

**Fig 5:**
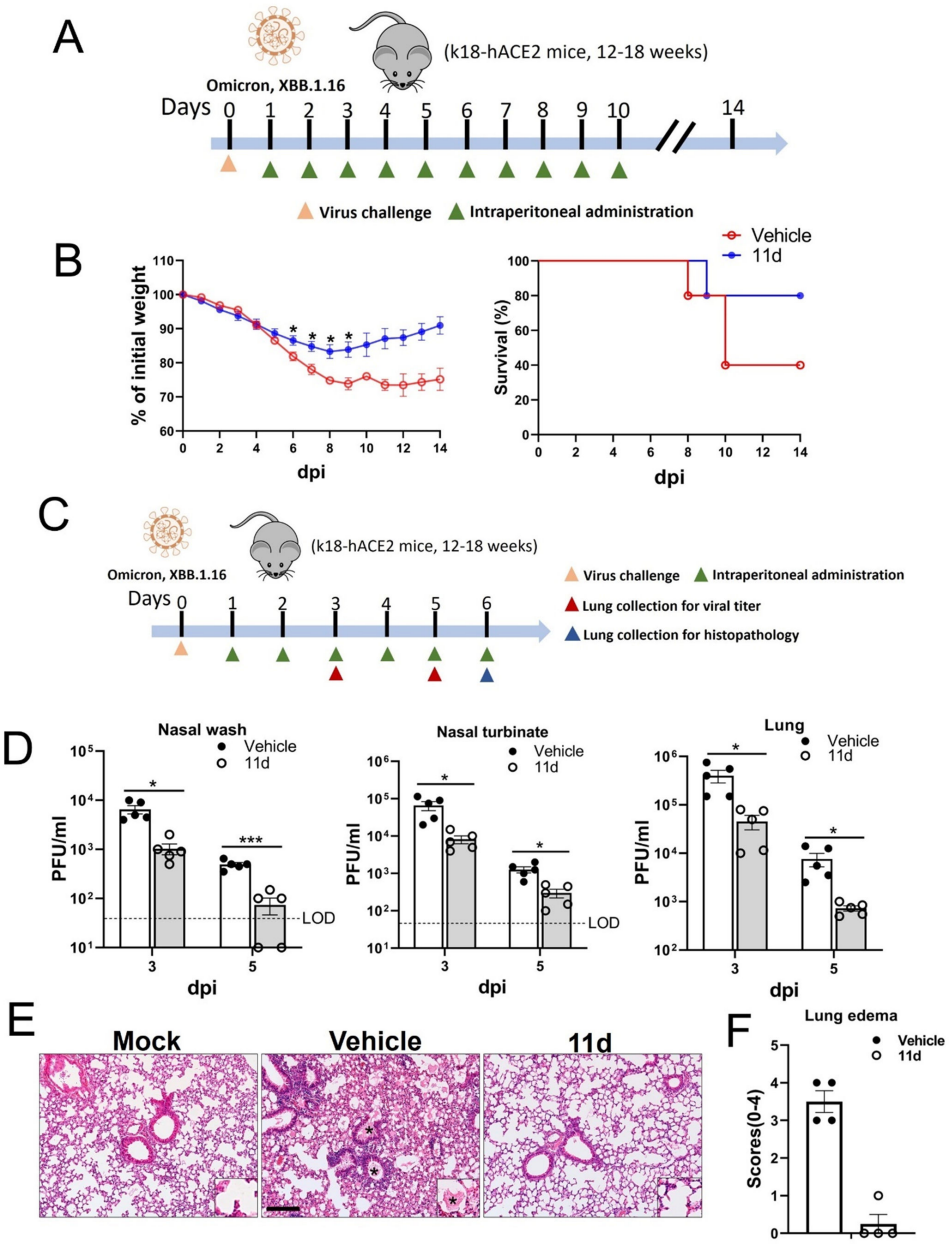
Post-infection treatment of ***11d*** in K18-hACE2 mice infected with SARS-COV-2 omicron variant. (**A–C**) Schematic (**A**) of the experimental procedure for post-infection treatment in K18-hACE2 mice with ***11d***. K18-hACE2 mice were intranasally inoculated with SARS-CoV-2 subvariant XBB.1.16 at 0 dpi. Infected mice were intraperitoneally administered with ***11d*** or vehicle from 1 to 10 dpi. Body weight (B, left) and survival (B, right) were recorded daily from 0 to 14 dpi (*n* = 5 each group). (**C–F**) Schematic of experiment (**C**) for testing the inhibitory activity of ***11d*** on omicron XBB.1.16 viral replication (**D**) and pathogenesis (**E**) in K18-hACE2 mice. At 3 and 5 dpi, infectious viruses from nasal wash, nasal turbinate, and lung were titrated (*n* = 5 each group) (**D**). Lungs were collected at 6 dpi for histopathologic examination (*n* = 4 each group) (**E**). Average scores of lung edema by vehicle or ***11d*** treatment (**F**). Bar indicates 170 µm. Data are presented as mean ± standard error of mean (SEM). Asterisks in B and D indicate statistical significance (*P < 0.05, **P < 0.01, ***P < 0.001).

We tested the antiviral ability of ***11d*** on SARS-CoV-2 Omicron subvariant XBB.1.16 in K18-hACE2 mice. In mice infected with the Omicron variant, treatment with vehicle led to 60% fatality by 14 days after virus infection, but compound ***11d*** treatment reduced the fatality to 20%. Vehicle-treated mice lost over 20% of weight at 7 dpi to the end. Mice treated with ***11d*** reduced weight losses compared to those treated with vehicle (Fig. 5B), showing significant difference at 5 to 9 dpi. The weight of ***11d*** treated mice started to recover from 8 dpi, but those treated with vehicle did not recover weight losses to 14 dpi (Fig. 5B). When assessed of viral loads in nasal wash, nasal turbinate, and lungs, viral titers from all tissues were significantly lower with ***11d*** treatment compared to those with vehicle treatment at 3 and 5 dpi (Fig. 5D). While severe edema (average score 3.5) and perivascular infiltrates were evident in the lungs from vehicle-treated animals, ***11d*** treatment led to significantly reduced lung pathologies with little edema (average score <0.5) (Fig. 5E and F).

## DISCUSSION

SARS-CoV-2 infections continue to have a significant impact on public health worldwide (2) with emergence of multiple variants (12, 44, 45) reducing the effectiveness of vaccines and monoclonal antibody therapy (46–48). Small-molecule inhibitors, Paxlovid (a combination of the 3CL^pro^ inhibitor nirmatrelvir and ritonavir, a CYP450 3A4 inhibitor), and the viral RNA-dependent RNA-polymerase inhibitors Molnupiravir and Remdesivir have been commercialized for COVID-19. Paxlovid is a strong inhibitor of CYP3A and contraindicated with drugs that are highly dependent on this enzyme for clearance (49), so the availability of additional direct-acting 3CLpro antivirals would be highly desirable as single or combination therapy. In addition, several 3CLpro inhibitors are in preclinical or clinical development and many of them have shown to be effective in experimentally infected (mice and hamsters) for SARS-CoV-2 or MERS-CoV infection (15, 17–28). Among them, oral protease inhibitor Ensitrelvir (27) is in clinical trials (Phase 3 as of April 2023).

In our study, we determined the dual roles of 3CLpro inhibitors against SARS-CoV-2 3CLpro and cathepsin enzymes, as well as post-infection efficacy of select compounds in fatal mouse models of SARS-CoV-2 or MERS-CoV infections. Studies of entry of SARS-CoV-2 into cells have shown that virus entry routes through the cell membrane are dependent on the expression of the host cell surface protease TMPRSS2, and virus entering cells lacking TMPRSS2 via the endosomal pathway, which requires endosomal enzymes, such as cathepsin L and B (31, 50). Therefore, compounds targeting multiple targets such as virus 3CLpro and cathepsins may be beneficial for antiviral activity and may limit antiviral resistance. One of our reported compounds, GC376, was previously shown to have dual activity against cathepsin (23, 41), and we determined that ***5c/d** and **11c/d*** have anti-cathepsin B and L activity with nanomolar IC_50_ values (Table 2). These compounds have potent activity against SARS-CoV-2 and MERS-CoV in enzyme assay and viral replication (SARS-CoV-2 replicon) (Table 1). The dual inhibitory effects of the compounds in cells are demonstrated in cells lacking expression of TMPRSS2, where virus entry is presumed to be dependent on the activity of cathepsins (Table 1). As most susceptible cells in the respiratory system express both hACE2 and TMPRSS2, inhibition of the viral endosomal route may not play a significant antiviral role in the major target tissue of SARS-CoV-2 in humans. However, SARS-CoV-2 has been reported to infect cells with no or little TMPRRS in other organs, including the intestines with clinical effects (51, 52), and it remains to be determined whether this dual effect offers clinical benefits.

SARS-CoV-2 and MERS-CoV, both betacoronaviruses, belong to different subgenera, *Sarbecoviru*s and *Merbecovirus*, respectively. The homology of the amino acid sequences of SARS-CoV-2 and MERS-CoV 3CLpros used in this study is 50.83%, but the overall structures of 3CLpros are highly conserved among various coronaviruses with a unique primary substrate specificity for a P1 Gln residue. Molecular docking studies based on our previous co-crystallographic structures of SARS-CoV-2 and MERS-CoV 3CLpros bound with series of compounds related to *11*c (41) showed *11*c adopts a similar binding mode to SARS-CoV-2 and MERS-CoV 3CLpro with minor differences (Fig. 1), which may explain the similar IC_50_ values against both coronavirus 3CLpro (Table 1).

Transgenic mice expressing hACE2 and non-transgenic mouse models for SARS-CoV-2 infection have widely been used to study SARS-CoV-2 infection and pathogenesis and evaluation of countermeasures against SARS-CoV-2 infection (53–55). SARS2-N501Y_MA30_ carries multiple adapted amino acid changes after being passaged in mice lungs and can infect non-transgenic mice, such as BALB/c, causing fatal infection (56). Transgenic hDPP4-KI mice have been excellent models for MERS-CoV infection with respiratory signs and fatality after virus inoculation (57–59). Further adaptation of MERS-CoV following serial passages in mice increased the virulence in hDPP4-KI mice (58). We have recently reported 3CLpro inhibitors that are highly effective against MERS-CoV and SARS-CoV-2 in animal models (28, 38). In mice infected with MA-MERS-CoV, treatment with *6*j starting one or two days after virus infection significantly increased survival and reduced lung viral titers and histopathology (28). In another report, we demonstrated *in vivo* efficacy of a deuterated derivative of GC376 against SARS-CoV-2 using K18-hACE2 mice (38). In this report, we tested the efficacy of ***5d*** and ***11d*** in mice to demonstrate their broad-spectrum activity against these two important coronavirus infections. While ***5d*** and ***11d*** show similar *in vitro* potency against both coronaviruses (Table 1), and both compounds significantly increased survival of mice when treatment started 1 day post infection compared to no-treatment, ***11d*** had a greater impact on reducing fatality and body weight changes than ***5d**.* Analyses of lung viral loads and histopathological changes as well as levels of pro-inflammatory cytokines including CXCL-10, IL-6, and TNF-α in infected mice treated with ***11d*** revealed significantly reduced titers, fewer histopathological changes, and reduced proinflammatory cytokine levels compared to no-treatment, which correlate well with the observed improvement in survival. *In vivo* efficacy is influenced by many factors, including bioavailability, PK, metabolism, and the chemical stability of a compound, which may explain the relatively lower efficacy of 5d in mice.

Because current predominant circulating SARS-CoV-2 are Omicron variants (60), we examined ***11d*** in K18-hACE2 mice infected with SARS-CoV-2 Omicron XBB.1.16. It has been shown that SARS-CoV-2 Omicron variants are known to cause less severe diseases in the mouse model (45, 61, 62). Because infections of Omicron subvariant XBB.1.16 in K18-hACE2 mice can lead to fatal outcome, we used this combination to evaluate *in vivo* efficacy of ***11d*** in this study. Inoculation of Omicron XBB.1.16 in K18-hACE2 mice with 2 × 10^4^ PFU resulted in up to 60% fatality by 14 dpi with vehicle-treatment. The treatment with ***11d*** showed that similar results (Fig. 5) seen in BALB/c mice infected with MA-SARS-CoV-2 or hDPP4-KI mice infected with MA-MERS-CoV, which further confirmed *in vivo* potency of ***11d*** in the models.

In summary, we show that the tested 3CLpro inhibitors have anti-coronavirus (SARS-CoV-2 and MERS-CoV) protease and cathepsin B/L activities and inhibitor ***11d*** significantly enhances survival of mice fatally infected with SARS-CoV-2 or MERS-CoV when treatment started 1 day post infection. While there are several reports of 3CLpro inhibitors effective against human coronaviruses in animal models, to the best of our knowledge, this is the first report demonstrating a single compound being highly effective in both fatal animal models of SARS-CoV-2 (both MA strain and Omicron variant) and MERS-CoV treated after viral infections. While we demonstrated *in vivo* efficacy of compound ***11d*** using the mouse models, clinical efficacy is influenced by many factors including host-specific components, which poses a major challenge in drug development, and further research is needed to establish whether these inhibitors can be effective therapeutics in humans. Nevertheless, our results add to the current arsenal of potential protease inhibitors of SARS-CoV-2 and MERS-CoV.

## MATERIALS AND METHODS

### Study design

The primary objective of this study was to further evaluate the antiviral activity of compounds ***5d*** and ***11d*** (41) in fatal mouse models of SARS-CoV-2 (56) or MERS-CoV infection (57, 58). Compounds ***5d*** and ***11d*** were also evaluated for efficacy against several human proteases, including cathepsin L, using lentivirus-based pseudotyped viruses containing SARS-CoV-2 S in cells expressing human angiotensin-converting enzyme-2 (hACE2) or hACE2 plus TMPRSS2.

### Cells, viruses, and compounds

Vero E6, Vero-ACE2-TMPRSS2, Huh-7, Vero 81, and 293T cells were maintained in Dulbecco’s minimal essential medium (DMEM) containing 10% fetal bovine serum (FBS) and antibiotics [chlortetracycline (25 µg/mL), penicillin (250 U/mL), and streptomycin (250 µg/mL)]. Calu-3 2B4 cells were grown in DMEM supplemented with 20% FBS and antibiotics. MA-SARS-CoV-2 (SARS2-N501Y_MA30_) (56) and MA-MERS-CoV (57) were propagated in Calu-3 2B4 and Huh-7, respectively. SARS-CoV-2 XBB.1.16 omicron variant (isolate hCoV-19/USA/CA-Stanford-139_S23/2023) was obtained from BEI. Lentivirus-based pseudoviruses expressing SARS-CoV-2 Wuhan-Hu-1 strain were generated with the second-generation lentiviral packaging plasmid psPAX2 (Addgene, Watertown, MA), a reporter plasmid pUCGFP-Luc (Addgene), and pAbVec-SARS2-S (42). Both plasmids expressing hACE2 or TMPRSS2 were obtained from Origen (Rockville, MD). MDL28170 (calpain and cathepsin B inhibitor) and Nafamostat (TMPRSS2 inhibitor) were purchased from Sigma-Aldrich (St Louis, MO). SARS-CoV-2 3CLpro inhibitors, ***5c/d*** and ***11c/d*** were recently reported from our labs (41). Polybrene was purchased from Santa Cruze Biotechnology (Santa Cruz, CA).

### Activity of *5c/d* and *11c/d* on SARS-CoV-2 pseudovirus entry assays in cells

To elucidate the potential dual roles of ***5c/d** and **11c/d*** in the entry of SARS-CoV-2, we used lentivirus-based pseudotyped viruses expressing coronavirus S proteins (42). In addition to ***5c/d** and **11c/d***, well-known cathepsin L aldehyde inhibitors, including MDL28170 and a trypsin inhibitor, Nafamostat were tested in the virus entry assay. The virus entry assay was performed in 293T cells expressing hACE2 alone or hACE2 plus TMPRSS2 was previously established in our lab (42). Briefly, cells were incubated with serial dilutions of ***5c/d**, **11c/d**,* MDL28170, Nafamostat, or DMSO (mock) and immediately transduced with pseudotyped virus in the presence of polybrene (10 µL/mL). The dose-dependent inhibition curve for each compound was prepared and the EC_50_ values were determined by GraphPad Prism software using a variable slope (GraphPad, La Jolla, CA).

### Activity of 5c/d and 11c/d against human proteases

The 50% inhibitory concentration (IC_50_s) of ***5c/d*** and ***11c/d*** against SARS-CoV-2 3CLpro or MERS-CoV 3CLpro and the 50% effective concentration of (EC_50_) against SARS-CoV-2 replicon were reported in our recent paper (41). The IC_50_s of ***5c/d** and **11c/d*** against human proteases, including thrombin (AnaSpec, Fremont, CA), chymotrypsin (Sigma-Aldrich, St Louis, MO), neutrophil elastase (Sigma-Aldrich), cathepsin B (Abcam, Eugene, OR), cathepsin D (Abcam), cathepsin G (Novus Biological, Englewood, CO), and cathepsin L (AnaSpec) were determined using commercial enzyme assays following manufacturer’s procedures.

### Modeling of the *11c* binding mode with SARS-CoV-2 and MERS-CoV 3CLpro

The binding modes of *11*c (aldehyde form of ***11d***) in the 3CLpro active sites of SARS-CoV-2 and MERS-CoV were modeled using previously determined crystal structures (41) of each 3CLpro. The binding mode of *11*c in the active sites was modeled using the coordinates of previously determined crystal structures of SARS-CoV-2 3CLpro [PDB 7TQ5 (41)] and MERS-CoV 3CLpro [PDB 5WKK (63)] superimposing the inhibitor in the active sites. Superposed models of *11*c were prepared for docking by adding the covalent bond between the inhibitor and the Sγ atom of the active site Cys, specifying His163 (SARS-CoV-2) and His166 (MERS-CoV) as the HIE tautomer with protonation of the Nε atom. The protein preparation wizard in Schrodinger was used to optimize hydrogen bonding and minimize the structure using Schrodinger’s OSPL4 energy function (64) and *11*c was prepared for docking using LigPrep (64). These models were subsequently used for covalent docking using CovDock, also from Schrodinger (64, 65), selecting the “Nucleophilic Addition to a Double Bond” reaction, adding a core constraint to the glutamine surrogate side chain, performing MM-GBSA scoring, and outputting five poses per ligand reaction site. The hydroxyl formed after covalent attachment to Cys145 was oriented toward His41 and the complex was minimized allowing for flexibility within 6 Å of the ligand while constraining the key hydrogen bonds that are typically observed in the P1 and P2 sites.

### Biocontainment and biosafety of coronaviruses

All *in vitro* studies with live SARS-CoV-2 were performed in biosafety level 3 facilities at the University of Iowa, and the studies with SARS-CoV-2 replicon and lentivirus-based pseudoviruses were performed in biosafety level 2 facilities at Kansas State University under protocols approved by the Institutional Biosafety Committee at the University of Iowa and Kansas State University, respectively, according to the guidelines set by the Biosafety in Microbiological and Biomedical Laboratories, the U.S. Department of Health and Human Services, the U.S. Public Health Service, the U.S. Centers for Disease Control and Prevention, and the National Institutes of Health. *In vivo* studies were performed in animal biosafety level 3 facilities at the University of Iowa. All mouse experiments were conducted under protocols approved by the Institutional Animal Care and Use Committee at the University of Iowa according to the guidelines set by the Association for the Assessment and Accreditation of Laboratory Animal Care and the U.S. Department of Agriculture.

### Post-infection treatment in MA-SARS-CoV-2 infected mice

Compounds ***5d*** and ***11d*** were examined for efficacy using 10-weeks-old female BALB/c mice infected with MA-SARS-CoV-2 (SARS2-N501Y_MA30_) (56). For evaluating body weight and survival rates, animals were divided into three groups (*N* = 3 for vehicle or *N* = 5 for compound ***5d*** or ***11d***) and were lightly anesthetized with ketamine/xylazine prior to infection with 50 µL of 1,000 PFU MA-SARS-CoV-2 via intranasal inoculation. Both compounds were formulated in 10% ethanol and 90% PEG400 and given to mice from 1 (24 h post infection) to 10 days post infection (dpi) at 100 mg/kg/day (once per day) via intraperitoneal administration. Control mice received vehicle. Animals were weighed daily and monitored for 14 days. The same experiment was repeated, and results were combined (thus, total *N* = 6 for vehicle or *N* = 10 for compound ***5d*** or ***11d***). To evaluate virus replication in lungs, 10-weeks-old BALB/c mice were divided into 4 groups (*N* = 5 for vehicle or ***11d*** at 3 or 5 dpi) and infected with 50 µL of 1 × 10^3^ PFU MA-SARS-CoV-2 via intranasal inoculation. Compound ***11d*** was given to mice from 1 (24 h post infection) to 3 or 5 dpi at 100 mg/kg/day (once per day) via intraperitoneal administration. Animals were euthanized when an animal lost 30% of its’ initial weight or at 14 dpi, and the lungs were harvested to evaluate for virus titration. One animal in the vehicle group died at 5 dpi. SARS-CoV-2 virus titration was done in Vero E6 cells using a plaque assay, and mRNA levels of viral N gene were quantified by RT-qPCR.

An additional experiment was conducted to evaluate histopathological changes in the lungs of mice treated with vehicle or compound ***11d*** (*N* = 5). Animals were euthanized at 5 dpi, and the lungs were fixed with 10% formalin. Hematoxylin and eosin (HE) stained lung tissues were examined by a veterinary pathologist (DKM) using the post-examination method of masking (66). Lung tissues were evaluated for edema (0–4) using distribution-based ordinal scoring: 0, none; 1, <25% of field; 26%–50% of field; 51%–75% field and >75% of field.

### Post-infection treatment in mice infected with MERS-CoV infection

Compounds ***5d*** and ***11d*** were also examined using 13- to 15-weeks-old hDDP4-KI mice infected with MA-MERS-CoV (57). For evaluating body weight and survival rates, animals were divided into three groups (*N* = 4 for vehicle, *N* = 4 for ***5d*** or ***11d***) and were lightly anesthetized with ketamine/xylazine prior to infection with 50 µL of 800 PFU of MA-MERS-CoV via intranasal inoculation. Treatment schedule and duration were the same as for the SARS-CoV-2 studies. The same experiment was repeated, and the results were combined (thus, total *N* = 8 for vehicle, *N* = 8 for ***5d*** or ***11d***). To evaluate virus replication in lungs, 13- to 15-weeks-old hDDP4-KI mice were divided into 4 groups (*N* = 5 for vehicle or ***11d***) and infected with 50 µL of 800 PFU MERS-CoV via intranasal inoculation. Treatment schedule and duration were the same as for the SARS-CoV-2 studies. MERS-CoV virus titration was detected in Vero 81 cells using a plaque assay, and RT-qPCR was used to quantify viral N gene expression. Histopathology in the lungs of mice treated with vehicle (*N* = 4) or compound ***11d*** (*N* = 5) was examined as described above for SARS-CoV-2. Animals were euthanized at 6 dpi, and lung tissues were evaluated using the same criteria as SARS-CoV-2 studies.

### Post-infection treatment in K18-hACE2 mice infected with SARS-CoV-2 Omicron variant

Compound ***11d*** was examined to test its antiviral ability against Omicron subvariant XBB.1.16 in K18-hACE2 mice. Twelve- to eighteen-weeks-old K18-hACE2 mice were divided into two groups (*N* = 5 for vehicle or ***11d*** group) and were lightly anesthetized with ketamine/xylazine prior to intranasally challenging with 50 µL of 2 × 10^4^ PFU of Omicron XBB.1.16. Treatment schedule and duration were similar to the SARS-CoV-2 or MERS-CoV studies as shown in Fig. 5. Omicron XBB.1.16 from nasal washes, nasal turbinates, and lungs were collected at 3 and 5 dpi and titrated using the plaque assay in Vero-ACE2-TMPRSS2 cells. Lung histopathological analysis was performed as above-mentioned in SARS-CoV-2 studies.

### Viral titer by plaque assay

Viruses from lung homogenates were serially diluted in DMEM. Indicated cells in the 12 well plates were inoculated with 200 µL of diluted viruses for 1 h and rocked every 15 min. After removing the viruses, cells were overlaid with 0.6% agarose containing 2% FBS. After 3 days, infected cells were fixed with 10% formaldehyde, and plaques were counted after staining with 0.1% crystal violet.

### Real-time quantitative qPCR

Total RNA was isolated from mouse lungs using TRIzol (Invitrogen) according to manufacturer’s protocol. One microgram RNA was subjected to reverse transcription reaction. The resulting cDNA was used to assay the expression of the indicated mRNA. Housekeeping gene hypoxanthine-guanine phosphoribosyltransferase (HPRT) was used as internal control. The relative abundance of each gene was normalized to HPRT and presented as 2^−Δ*Ct*^. The following RT-QPCR primers were used in this study:

SARS-CoV-2 N-forward: 5′-GACCCCAAAATCAGCGAAAT-3′;
SARS-CoV-2 N-reverse: 5′-TCTGGTTACTGCCAGTTGAATCTG-3′.
MERS-CoV N-forward: 5′-GGCACTGAGGACCCACGTT-3′;
MERS-CoV N-reverse 5′-TTGCGACATACCCATAAAAGCA-3′.
Mouse HPRT forward: 5′-GCGTCGTGATTAGCGATGATG-3′;
Mouse HPRT reverse: 5′-CTCGAGCAAGTCTTTCAGTCC-3′.
Mouse TNF-α forward: 5′-GAACTGGCAGAAGAGGCACT-3′;
Mouse TNF-α reverse: 5′-AGGGTCTGGGCCATAGAACT-3′.
Mouse IL-6 forward: 5′-GAGGATACCACTCCCAACAGACC-3′;
Mouse IL-6 reverse: 5′-AAGTGCATCATCGTTGTTCATACA-3′.
Mouse CXCL-10 forward: 5′-GCCGTCATTTTCTGCCTCAT-3′;
Mouse CXCL-10 reverse: 5′-GCTTCCCTATGGCCCTCATT-3′.

### Statistical analysis

Statistical differences between indicated groups for mice body weight changes, viral loads, and histopathological score were analyzed using two-tailed unpaired *t* test with Welch’s correction. Log-rank (Mantel-Cox) test was used for the analysis of survival curves between groups. All statistical analyses were performed in GraphPad Prism 8.0 software (San Diego, CA). *P* < 0.05 was considered statistically significant. *P < 0.05, **P < 0.01, ***P < 0.001.
